# Complex PTSD in Digital Age: Case of Ukrainian Refugees – Victims of War Crimes

**DOI:** 10.1192/j.eurpsy.2025.1955

**Published:** 2025-08-26

**Authors:** M. Jishkariani, N. Okribelashvili, G. Berulava

**Affiliations:** 1 Clinical Department, Psycho - Rehabilitation Centre “EMPATHY”; 2 Psychiatry, TSU, Tbilisi, Georgia

## Abstract

**Introduction:**

**This study includes the characteristics of Compex PTSD among Ukrainian IDPs who experienced the horrors of the war - during the Russia-Ukraine war in 2022-2024. It also reflects the new and permanent stressors characteristic of the digital age**.

**Objectives:**

Objective of the Study was to identify how cyber age and modern technologies affected on outcomes of traumatic stress.

**Methods:**

**The study was carried out iamong the victims of the Ukrainian war temporarily displaced in Georgia. A specially developed questionnaire based on the guidelines of the Istanbul Protocol was used.**

**Results:**

A total number of direct target beneficiaries applied to the Empathy was n = 68 persons; Most refugees were with experience of war crimes. **Mainly two types of life threatening traumatic events was identified:** Locally in place of war and Russian occupation; They flowed from Ukraine through the Russian territory and additionally are with experience of ill – treatment during the filtration procedures in the so-called “filtration camps”. Chronic Repetitive Traumatic Stressors were identified as well: Misinformation; Adhering to online war scenes, the vast amount of information flow; Inability to dissociate from warfare. Observed: Rapid development of C - PTSD in 52 persons of total 68. Alongside with PTSD symptoms reveled: Affective Dysregulation – aggression, self – aggression, depression; Feeling of Guilty – for not being in Ukraine, not supporting to their relatives and Country; Problems in interpersonal relations; Addictive behavior; Additional stressors related to the digital warfare and disinformation supported the prolongation of multi natural stressors that consequently created significant problems for recovery.

**Conclusions:**

**The results were discussed in the context of Carl Jaspers’ conception regarding reactive conditions.** According to his concept, reactive psychoses develop on the basis of a conflict between a person and an unbearable reality for him, and at the same time, this reality is necessarily reflected in the clinical picture of the disease. However, it should be noted that by the 60s, delayed forms of reactive states were described, i.e., forms far removed from the traumatic moment, during which Jasper’s criterion of “time connection” is violated, although the traumatic event is fully reflected in the clinical picture. The reason for this is mainly the prolonged process of intrapsychic processing and final realization of the trauma. **The rapid development of complex PTSD under the combined influence of endless multi-stress factors was revealed; The researchers were faced with an effective treatment - the search for ways of rehabilitation in the current multi-stress conditions. Furthermore studies have to be addressed to create new complex recovery strategies in modern multi – face unsolved stress situations.**

**Disclosure of Interest:**

None Declared

